# Synergistic Inhibitory Effect of Honey and *Lactobacillus plantarum* on Pathogenic Bacteria and Their Promotion of Healing in Infected Wounds

**DOI:** 10.3390/pathogens12030501

**Published:** 2023-03-22

**Authors:** Mei Li, Hong Xiao, Yongmei Su, Danlin Cheng, Yan Jia, Yingli Li, Qi Yin, Jieying Gao, Yong Tang, Qunhua Bai

**Affiliations:** 1 Department of Health Laboratory Technology, School of Public Health, Chongqing Medical University, Chongqing 401334, China; 2 Chongqing College of Traditional Chinese Medicine, Chongqing 402760, China; 3 The First Clinical School, Chongqing Medical University, Chongqing 400016, China; 4 Chongqing Orthopedics Hospital of Traditional Chinese Medicine, Chongqing 400039, China

**Keywords:** honey, *Lactobacillus plantarum*, pathogenic bacteria, antibacterial, biofilm, wound infection

## Abstract

Prevention and control of infections have become a formidable challenge due to the increasing resistance of pathogens to antibiotics. Probiotics have been discovered to have positive effects on the host, and it is well-known that some *Lactobacilli* are effective in treating and preventing inflammatory and infectious diseases. In this study, we developed an antibacterial formulation consisting of honey and *Lactobacillus plantarum* (honey–*L. plantarum*). The optimal formulation of honey (10%) and *L. plantarum* (1 × 10^9^ CFU/mL) was used to investigate its antimicrobial effect and mechanism in vitro, and its healing effect on wound healing of whole skin infections in rats. Biofilm crystalline violet staining and fluorescent staining results indicated that the honey–*L. plantarum* formulation prevented the biofilm formation in *Staphylococcus aureus* and *Pseudomonas aeruginosa* and increased the number of dead bacteria in the biofilms. Further mechanism studies revealed that the honey–*L. plantarum* formulation may inhibit biofilm formation by upregulating biofilm-related genes (*icaA*, *icaR*, *sigB*, *sarA*, and *agrA*) and downregulating quorum sensing (QS) associated genes (*lasI*, *lasR*, *rhlI*, *rhlR*, and *pqsR*). Furthermore, the honey–*L. plantarum* formulation decreased the number of bacteria in the infected wounds of rats and accelerated the formation of new connective tissue to promote wound healing. Our study suggests that the honey–*L. plantarum* formulation provides a promising option for the treatment of pathogenic infections and wound healing.

## 1. Introduction

Bacterial infectious diseases pose a major threat to human health as they cause a significant burden of morbidity and mortality worldwide. Among common infectious diseases, *Staphylococcus aureus* (*S. aureus*) and *Pseudomonas aeruginosa* (*P. aeruginosa*) are the most common causative organisms detected in chronic wounds [1]. Usually, *S. aureus* is located on the surface of the infection and *P. aeruginosa* is located deeper in the infection [2] Because these bacteria have a high level of antibiotic resistance, treating wound infections can be difficult. Additionally, both bacteria produce biofilms, have high resistance to many antimicrobial medications [3,4], and are the leading causes of most chronic infections [5]. Pharmacological treatment for chronic wound infections involves the systemic administration of antibiotics. Still, the development of drug resistance has made more and more antibiotics lose their effectiveness of anti-infection. Various coping strategies have been investigated, particularly drug designs based on synthetic analogs that can inhibit virulence factors. However, these studies have not yielded promising results due to toxicity and low bioavailability. There remains a need to develop alternative therapies to manage wound bacterial infections effectively.

Lactic acid bacteria (LAB), a group of bacteria that use carbohydrates to produce lactic acid, have been used for thousands of years to ferment and preserve food [6]. They naturally control the microbial composition of many foods because these lactic acid bacteria have antagonistic and inhibitory properties by competing for nutrients or producing active antimicrobial metabolites such as organic acids, hydrogen peroxide, acetylacetone, diacetyl, and bacteriocins [7]. *Lactobacilli* can reduce the risk of infectious diseases, fight secondary infections with antibiotics [8], and reduce antibiotic therapy’s incidence and severity of diarrhea [9]. In addition, most *Lactobacilli* are generally considered safe by the US Food and Drug Administration [10]. *Lactobacillus plantarum* (*L. plantarum*) is one of the typical representatives of lactic acid bacteria, and topical application of *L. plantarum* has been demonstrated to reduce or eliminate the pathogenic bacterial load, reduce necrotic tissue, accelerate the appearance of granulation tissue, reduce wound area, and promote wound healing [11]. Moreover, topical administration of *L. plantarum* accelerated the healing of chronic diabetic foot ulcers and infected burn wounds by altering infection, angiogenesis, macrophage phenotype, and neutrophil response [12,13]. It was also found that *L. plantarum* supernatant has protective effects against bacterial infection, oxidative stress, and wound healing [14,15]. According to the studies mentioned above, probiotics and their metabolites may be able to treat bacteria that are resistant to antibiotics. To combat the significant worldwide danger posed by antimicrobial resistance, future research should be conducted on creating combinations of probiotics and their metabolites, which are promising alternatives to antibiotics to treat drug-resistant bacteria.

For many years, honey has been used as a folk medicine to treat wound infections. Honey’s healing and antimicrobial activity are partly attributed to its hygroscopic properties, high osmotic pressure, low pH, and hydrogen peroxide content [16,17,18]. A *Lactobacillus* (LAB) symbiotic community of nine *Lactobacillus* species and four *Bifidobacterium* species had been discovered in honeybee crops. Interestingly, these *Lactobacillus* symbionts have a role in the honey formation and are abundant in fresh honey. In addition, it was shown that these symbionts create a variety of extracellular proteins, including enzymes, bacteriocins, and lysozymes, as well as anti-microbial compounds such as formic acid, hydrogen peroxide, and free fatty acids [19,20]. *Lactobacillus* symbiosis is involved in honey production and plays a vital role in the antimicrobial action of honey by producing numerous antimicrobial metabolites and peptides [20].

Research has been conducted on the mechanisms of probiotic antibacterial action, although these studies have mostly looked at probiotic metabolites and their related active substances, such as bacteriocins [7]. Probiotics can produce metabolites and potent antibacterial capabilities, but little study has been carried out on how they interact with other agents to impact how they inhibit pathogenic bacteria. Although previous studies have reported the combined antibacterial effect of *L. plantarum* and honey, these studies only focused on single pathogenic bacteria and did not explore the synergistic effect of honey and *L. plantarum* in more depth and comprehensively. Therefore, in the present study, our main objective was to determine the inhibitory and antibacterial mechanisms of honey and *L. plantarum* against different species of pathogenic bacteria and their healing effect on infected wounds.

Many cell surface and secreted virulence factors are linked to the development of *S. aureus* biofilms, and the cell surface virulence factor polysaccharide intercellular adhesion protein (PIA) plays a key role in promoting adhesion contacts between bacterial cells [21,22]. Regulation of *ica ADBC* expression is mediated by a number of proteins, including *sarA* and *sigB* as well as *IcaR*, and it has been demonstrated that this regulation is necessary for biofilm formation [21,21,23]. Expression of *sarA* and *agr* has been shown to play a central role in the regulatory circuit of *S. aureus*, which includes important but often opposing roles in biofilm formation. It has been shown that acute virulence factors are regulated by bacterial intercellular communication mechanism quorum sensing (QS) systems [24,25,26], which consist of the acyl-homoserine lactone (AHL)-dependent *las* and *rhl* systems [27]. Therefore, we will also explore the relationship between the expression of honey–*L. plantarum* formulation and *lasI*, *lasR*, *rhlI*, *rhlR,* and *pqsR* genes.

## 2. Materials and Methods

### 2.1. Bacterial Strains and Materials

*L. plantarum* purchased from the China General Microbial Strain Collection Management Center (CGMCC 1.12974) was seeded in De Man, Rogosa, and Sharpe (MRS) broth and incubated for 24 h at 37 °C in facultative anaerobic conditions. The bacterial culture was centrifuged at 10,000× *g* for 5 min at 4 °C, and the pellet was washed three times and re-suspended with Phosphate buffer solution (PBS) buffer at pH 7.2 before use. *P. aeruginosa* strain PAO1, *S. aureus*, and *E. coli* were selected as indicator strains and grown in Luria–Bertani (LB) broth overnight at 37 °C with 200 rpm before use. PA01 is a laboratory from the School of Public Health, Chongqing Medical University, China. *S. aureus* and *E. coli* are clinical specimens which were isolated from Chongqing Sixth People’s Hospital. The antibiotic resistance profile of *P. aeruginosa*, *S. aureus*, and *E. coli* strains is shown in Supplementary Table S1.

The honey used in this study is bacopa honey, which contains three main types of honey: rape honey, acacia honey, and wattle honey. The honey was sterilized by 25 k Gray Cobalt-source irradiation before use and is free of microbial components.

### 2.2. Optimal Antibacterial Formulation of Honey and L. plantarum

A multilevel experimental design was used to optimize the honey–*L. plantarum* formulation. In brief, honey content (X_1_) and *L. plantarum* concentration (X_2_) were set as two independent variables and there were three levels of each variable according to the results of the preliminary pre-experiment (Table 1). When different ratios of honey and *L. plantarum* acted together with *S. aureus* (1 × 10^8^ CFU/mL) for 12 h, the viable count of *S. aureus* (Y) was set as the dependent variable. Experimental trials were performed under all nine possible combinations, with three replicate experiments conducted simultaneously for each combination. Additionally, a blank control (containing only *S. aureus*, without honey and *L. plantarum*) was set. SPSS statistics analyzed the results to select the best antibacterial formulation for honey and *L. plantarum*.

### 2.3. In Vitro Antibacterial Activity of Honey–L. plantarum Formulation

The vitro antibacterial activity of honey–*L. plantarum* formulation against *S. aureus*, *P. aeruginosa,* and *E. coli* was evaluated. In brief, the honey–*L. plantarum* group (HL, pathogens + honey + *L. plantarum*), negative control (C, Pathogens), *L. plantarum* control (L, pathogens + *L. plantarum*), and honey control (H, pathogens + honey) were configured separately. Specifically, cells from overnight cultures were diluted in LB broth to achieve 1 × 10^8^ CFU/mL. Diluted cultures with honey–*L. plantarum* formulation selected from the previous step were added (1 mL/well) into 24-well microplates and incubated at 37 °C. To obtain these viable culturable indicator cells, samples were collected at 6 h, 12 h, 18 h, and 24 h after incubation and diluted to a suitable concentration with PBS before being seeded on the LB agar plate. The CFU of bacteria on the LB agar plate was counted after being incubated for 24 h at 37 °C.

### 2.4. Biofilm Formation Inhibition Assay

The antibiofilm potential of honey–*L. plantarum* formulation was assayed using 24 well microtiter plates as described in a previous study with minor modifications [28]. Briefly, honey–*L. plantarum* formulation was mixed with *S. aureus* (5 × 10^5^ CFU/mL) or *P. aeruginosa* (5 × 10^5^ CFU/mL) in LB broth containing 0.1% glucose (HL). In addition, PBS, *L. plantarum,* and honey added with the bacterial culture were used as negative controls (S/P), *L. plantarum* control (L and honey control (H), respectively. Each bacterial solution was added to a 24-well microplate and incubated at 37 °C for 24 h. After incubation, the wells were rinsed twice with PBS to remove planktonic and non-adhering cells. The surface-bound cells were stained with 1 mL of 0.1% crystal violet (CV) solution for 10 min, followed by washing with PBS and destaining with 30% glacial acetic acid. The biofilm biomass was quantified by measuring the intensity of dissolved CV using a spectrophotometer at OD 595 nm by the Enzyme Markers (Thermo Scientific, Waltham, MA, USA).

### 2.5. Live/Dead Bacterial Staining

The effects were investigated of honey–*L. plantarum* formulation on biofilms of *S. aureus* and *P. aeruginosa,* which were stained with the SYTO9/PI Live/Dead Bacterial Double Stain Kit (MK Bio, Beijing, China). Biofilms were cultured on glass coverslips placed in a 24-well microplate with the honey–*L. plantarum* formulation. After incubation at 37 °C for 24 h, the biofilm specimens were gently washed twice with PBS to remove planktonic cells. The biofilm was stained for 15 min with the staining solution containing 3 µL of mixed staining solution (1.62 mM SYTO9 and 10 mM PI) in 1000 µL of 0.9% NaCl solution treated in the dark at room temperature for 15 min. Finally, the coverslips were gently fixed on the clean glass slides and observed under a fluorescence microscope (Olympus, Tokyo, Japan). Live bacteria were stained green and dead bacteria were stained red.

### 2.6. Quantitative Real-Time PCR Analysis

The overnight culture of *S. aureus* and *P. aeruginosa* were treated without (S/P) or with *L. plantarum* (L), honey (H), and honey–*L. plantarum* formulation (HL) at 37 °C for 24, and the cells were collected by washing three times with sterile PBS. Then, the total RNA was extracted using the Simply P Total RNA Extraction Kit (BioFIux, Beijing, China) according to the manufacturer’s protocol. Then, RNAs were converted to cDNA using PrimeScript™ RT (Takara, Tokyo, Japan). The LightCycler^®^ System (Thermo Scientific, Waltham, MA, USA) was used to analyze the expression of biofilm-related genes for *S. aureus* (*icaA*, *icaR*, *sigB*, *sarA*, and *agrA*) *and P. aeruginosa* (*lasI*, *lasR*, *rhlI*, *rhlR*, and *pqsR*) for *P. aeruginosa* involved in the quorum sensing (QS) mechanism and biofilm formation. The CR reaction was performed at a predefined ratio using PCR Master Mix (SYBR Green kit, Takara, Japan). The qRT-PCR primers are displayed in Table 2, and the *rpoB* (*S. aureus*) and *GAPDH* (*P. aeruginosa*) were internal reference genes, as described earlier [29,30]. The relative expression levels were calculated using the relative quantitative (2^−ΔΔCt^) method [31].

### 2.7. Changes in the Growth of L. plantarum in the Formulation

In this part, changes in the growth of *L. plantarum* were evaluated to understand the antimicrobial mechanism of the formulation. The honey–*L. plantarum* group (HL) and *L. plantarum* group (L) from the step “Inhibition of *S. aureus*, *P. aeruginosa* and *E. coli* by honey–*L. plantarum* formulation” were incubated for 24 h. Then, the bacterial solution was diluted with PBS. Next, the diluted solution was coated with MRS agar plates, and the number of *L. plantarum* was counted after incubation at 37 °C for 24 h. On the other hand, 1 × 10^9^ CFU/mL *L. plantarum* (L) and 10% honey*–L. plantarum* (HL) was added to the LB broth medium, and the growth of *L. plantarum* in it was detected using a Bioscreen Automated Microbial Growth Analyzer (Bioscreen C, Oy Growth Curves AB, Helsinki, Finland).

### 2.8. Antibacterial Effect of Honey–L. plantarum Culture Supernatant

To further investigate the antibacterial effect of honey–*L. plantarum* formulation, we tested the growth inhibition of *S. aureus* and *P. aeruginosa* by the cell-free supernatant (CFS) of honey–*L. plantarum* cultures and the effect of honey–*L. plantarum* on the pH value of the medium. Firstly, 10% honey and 1 × 10^7^ CFU/mL *L. plantarum* were incubated in an MRS medium at 37 °C for 24 h. Then, the cultures were centrifuged (at 10,000× *g*, for 5 min at 4 °C) to extract the supernatant, and the pH of the medium was tested with a Mettler Toledo pH Mete. After filtering the supernatant through a 0.22-μm sterile membrane, it was (10–20%, *v*/*v*) added to LB containing 1 × 10^7^ CFU/mL of *S. aureus* or *P. aeruginosa* and mixed well (HL). Meanwhile, cultured in LB broth, *S. aureus* or *P. aeruginosa* were also set as negative controls (C). Finally, the effect of the supernatant on the growth curves of the two bacteria (incubated) was measured at 37 °C for 24 h with a Bioscreen Automated Microbial Growth Analyzer.

### 2.9. In Vivo Animal Experiment

#### 2.9.1. Wound Infection Model

Eight-week-old male Sprague–Dawley (SD) rats weighing 200 ± 20 g were bought from the Chongqing Medical University’s Animal Experiment Center. Rats were housed singly under standard conditions with food and water ad libitum. The experimental animal handling methods conformed to animal ethics standards and were approved by the Experimental Animal Ethics Committee of Chongqing Medical University.

The specific scheme of the experimental design is shown in Supplementary Figure S1. Twelve adult male Sprague–Dawley rats were randomly divided into two groups, six in each group: the control group (SA) and the treated group (HL). After one week of adaptation, the experiment followed previously reported methods with modifications [32,33]. After being anesthetized with 10% chloral hydrate (300 mg/kg), the back of each rat was shaved, depilated, and washed with 75% ethanol [34,35]. Following that, a circular wound with a diameter of 10 mm was created on the back of each rat, which was subsequently infected with 30 μL *S. aureus* (1 × 10^8^ CFU/mL). Blank control (PBS) and honey–*L. plantarum* (HL) was applied to the wounds of the control group (SA) and the treated group (HL) of rats separately after an hour of drying. Afterward, the wounds were covered with commercially available transparent film dressings and secured with medical tape. On the zeroth, first, third, and fifth days, the formulation was changed once a day, and the wound healing was photographed. The rats were euthanized after five days.

#### 2.9.2. Evaluation of the Antibacterial Effect of Honey–*L. plantarum* Formulation on the Wounds

The rats were euthanized on the 1st and 5th days after treatment to assess the formulation’s antibacterial effect. The skin tissue along the wound edge was collected and homogenized in 1 mL of PBS with a homogenizer. From that, the sample solution was diluted to the optimal concentration, and 100 μL of the diluted solution was placed on the Baird-Parker agar plate. The number of colonies on the Baird-Parker agar plates was counted after 24 h incubation at 37 °C.

#### 2.9.3. Histological Analysis

For histological examination, excised wound skin tissue on days 1, 3, and 5 was fixed in 4% paraformaldehyde for at least 24 h, dehydrated in a graded series of ethanol, followed by xylene, and embedded in paraffin. Tissue sections were obtained from the center of the excised skin tissue and cut into five μm thick sections. Skin sections were stained with hematoxylin and eosin (H&E) to assess granulation tissue formation and wound maturity. Images were acquired using an inverted light microscope (Olympus, Tokyo, Japan).

### 2.10. Statistical Analysis

All data were analyzed and graphed using SPSS Statistics 25 (Armonk, NY, USA) and Graph Pad Prism 7 software (GraphPad Software, Inc., La Jolla, CA, USA), with quantitative results expressed as mean ± standard error (SEM). Statistical comparisons were performed using a *t*-test and one-way ANOVA, followed by Tukey’s Multiple Comparison test as the post-hoc test. A significant difference is marked as * (*p* < 0.05), ** (*p* < 0.01), *** (*p* < 0.001) and **** (*p* < 0.0001).

## 3. Results

### 3.1. Optimal Antibacterial Formulation of L. plantarum and Honey to Inhibit S. aureus

Previous experimental results suggest that there may be a synergistic antibacterial effect of *L. plantarum* and honey on *S. aureus*. To find the optimal antibacterial formulation of *L. plantarum* and honey, a two-factor, three-level analysis factor design experimental protocol was designed to determine the optimal concentration of *L. plantarum* and honey against *S. aureus*. As represented in Table 3, by analysis of variance, the main effects (H and L) show the inhibition of *S. aureus* by different concentrations of honey and *L. plantarum*. In contrast, the interaction term (X_12_) shows how the response changed when the concentrations of honey and *L. plantarum* were varied simultaneously. The result showed that there was an interaction between honey (H) and *L. plantarum* (L) (interaction term: H × L, *p* < 0.0001). Therefore, the antibacterial effect of honey and *L. plantarum* against pathogenic bacteria is mainly considered by the interaction between the two. Table 4 demonstrates that when the honey concentration in the formulation was 10% and the *L. plantarum* concentration was 1 × 10^9^ CFU/mL, *S. aureus* growth was the least (had the maximum inhibition rate), indicating that this formula has the best effect on suppressing the growth of *S. aureus*.

### 3.2. Honey–L. plantarum Formulation Inhibited the Growth of S. aureus, P. aeruginosa, and E. coli

**Figure 1:**
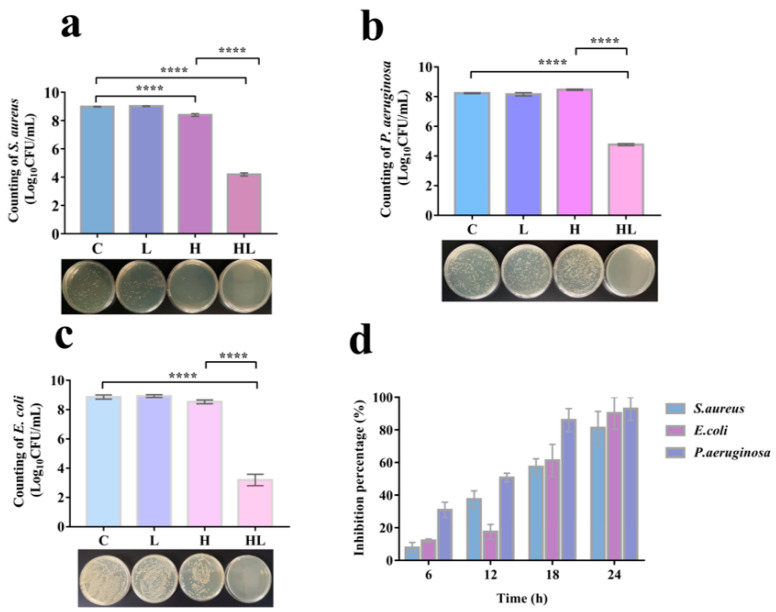
In vitro antibacterial activity of honey–*L. plantarum*. (**a**) inhibition of each component of the formulation against *S. aureus* (SA) for 12 h; (**b**) inhibition of each component of the formulation against *P. aeruginosa* (PA) for 12 h; (**c**) inhibition of each component of the formulation against *E. coli* (EC) for 12 h; (**d**) inhibition rate of the formulation against the three bacteria at different times. SA/PA/EC: 1 × 10^8^ CFU/mL; *L. plantarum*: 1 × 10^9^ CFU/mL; honey: (10%). Data are expressed as mean ± standard error, **** indicates a significant difference (*p* < 0.0001) compared with the blank (C) control.

Based on the results of pre-experiments, we hypothesized that honey and *L. plantarum* have a synergistic inhibitory effect on *S. aureus*. We tested the formulation’s ability to inhibit the growth of *S. aureus*, *P. aeruginosa*, and *E. coli* from understanding better the antibacterial activity of this formulation against pathogenic bacteria. The results are depicted in Figure 1a–c, where the honey–*L. plantarum* formulation can be seen after 12 h of interaction to have significantly inhibited the growth of these three bacteria. Moreover, of these bacteria, honey alone inhibited only *S. aureus*, with no effect on *P. aeruginosa* and *E. coli*. This again suggests that honey and *L. plantarum* have a synergistic antibacterial effect. Moreover, this antibacterial effect became stronger when the honey–*L. plantarum* formulation acted on these pathogenic bacteria longer. When the duration of action reached 24 h, the growth inhibition of *S. aureus*, *P. aeruginosa,* and *E. coli* by honey–*L. plantarum* formulation could reach more than 80% (Figure 1d).

### 3.3. Honey–L. plantarum Formulation Inhibited the Biofilm Formation of S. aureus and P. aeruginosa

**Figure 2:**
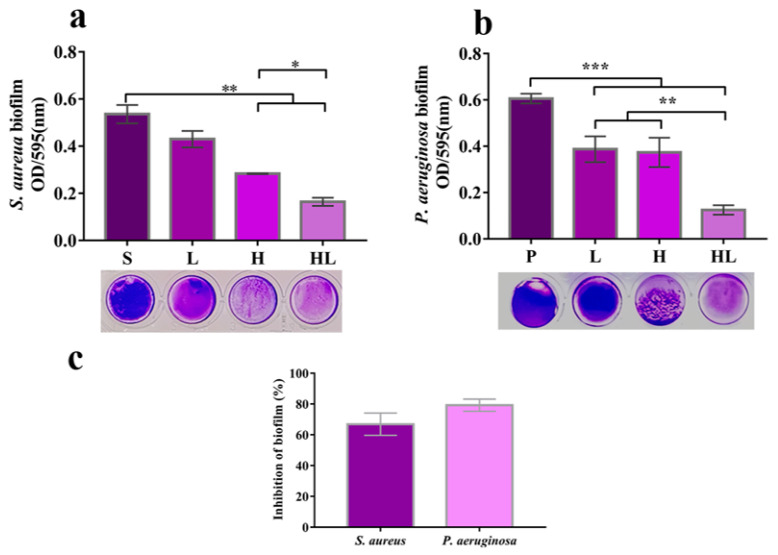
Effect of honey–*L. plantarum* formulation on biofilm formation. (**a**) Effect of honey–*L. plantarum* formulation on biofilm of *S. aureus*; (**b**) effect of honey–*L. plantarum* formulation on biofilm of *P. aeruginosa*. (**c**) Inhibition of biofilm formation of *S. aureus* and *P. aeruginosa* by formulations. S: *S. aureus* (1 × 10^8^ CFU/mL); P: *P. aeruginosa* (1 × 10^8^ CFU/mL); L: *S. aureus*/*P. aeruginosa* + *L. plantarum* (1 × 10^9^ CFU/mL); H: *S. aureus*/*P. aeruginosa* + honey (10% *v*/*v*); HL: *S. aureus/P. aeruginosa* + honey + *L. plantarum*. Data are expressed as mean ± standard error (SEM), * indicates a significant difference (*p* < 0.05) compared with the blank (S/P) control, ** indicates *p* < 0.01, and *** indicates *p* < 0.001.

To investigate whether the honey–*L. plantarum* formulation affects the biofilm formation of pathogenic bacteria, we measured the biofilm expression of *S. aureus* and *P. aeruginosa*. As shown in Figure 2, *L. plantarum* alone did not inhibit the biofilm formation of *S. aureus*. In contrast, honey and *L. plantarum* alone can inhibit the biofilm formation of *P. aeruginosa*, respectively. However, when honey and *L. plantarum* were combined, the inhibitory effect on biofilm formation was much better (*p* < 0.01). After the honey–*L. plantarum* formulation was added to the culture system for 24 h, the biofilm formation of *S. aureus* and *P. aeruginosa* were inhibited to 70% and 80%, respectively (Figure 2c). This indicates that honey and *L. plantarum* have a synergistic inhibitory effect on bacterial biofilm formation, which is consistent with the previous results that these two components synergistically inhibit the growth of these bacteria.

**Figure 3:**
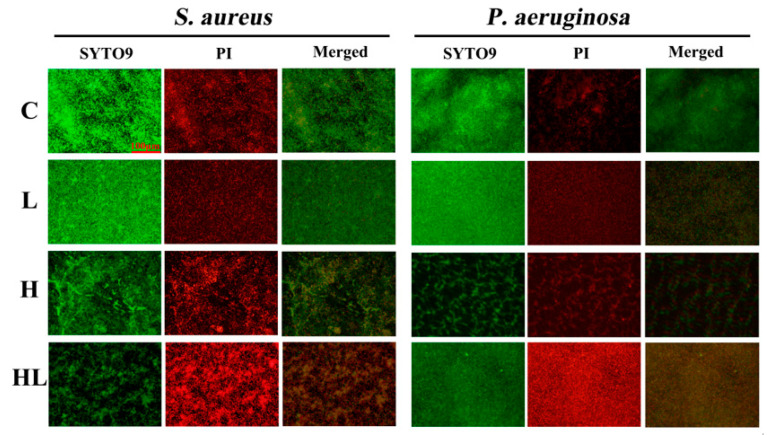
The effect of honey–*L. plantarum* formulation on biofilm formation of *S. aureus* and *P. aeruginosa* as observed by fluorescence microscope. C: *S. aureus* (1 × 10^8^ CFU/mL)/*P. aeruginosa* (1 × 10^8^ CFU/mL); L: *S. aureus*/*P. aeruginosa* + *L. plantarum*; H: *S. aureus/P. aeruginosa* + honey; HL: *S. aureus/P. aeruginosa* + honey + *L. plantarum*. Live cells were shown in green and dead cells in red, and ×20 magnification was used to observe.

A fluorescence microscope was applied to visualize the live and dead cells of the biofilms of *S. aureus* and *P. aeruginosa*. As shown in Figure 3, the living cells were labeled with SYTO9 (green fluorescence), whereas dead cells were stained with propidium iodide (red fluorescence). In the absence of the honey–*L. plantarum* formulation, the living cells appeared to be relatively intensive, and only a few dead cells could be observed in *S. aureus* and *P. aeruginosa* biofilms (C). Moreover, with *L. plantarum* treatment alone, the activity of the biofilm cells did not change, which could be observed by the increase in living cells and the few dead cells (L). However, many dead cells appeared in the field of vision when the biofilms were treated with honey alone (H) and honey–*L. plantarum* formulation (HL).

### 3.4. Honey–L. plantarum Formulation Increased the Transcription Level of icaA, icaR, sigB, sarA, and agrA, and Decreased the Transcription Level of lasI, lasR, rhlI, rhlR, and pqsR

**Figure 4:**
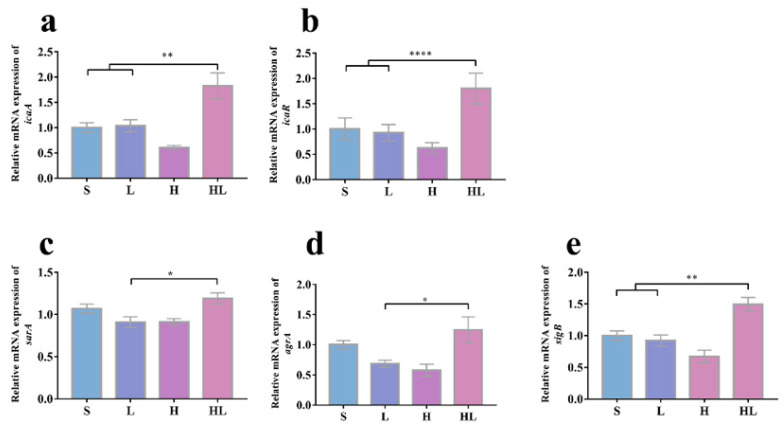
The effects of honey–*L. plantarum* formulation on the expression levels of *S. aureus* biofilm-related genes. Relative mRNA expression of *icaA* (**a**), *icaR* (**b**), *sarA* (**c**), *agrA* (**d**), and *sigB* (**e**). S: *S. aureus* (1 × 10^8^ CFU/mL); L: *S. aureus* + *L. plantarum*; H: *S. aureus* + honey; HL: *S. aureus* + honey + *L. plantarum*. Values are represented as mean ± standard error (SEM) (n = 3). The * indicates a statistically significant difference (*p* < 0.05), ** indicates *p* < 0.01, and **** indicates *p* < 0.001.

**Figure 5:**
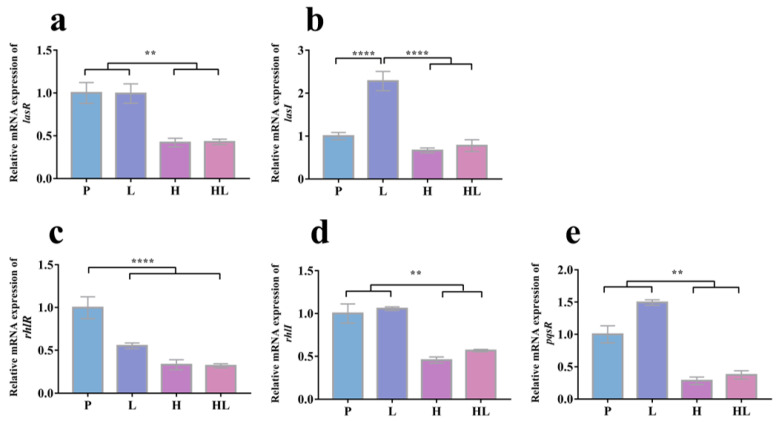
The effects of honey–*L. plantarum* formulation on the expression levels of *P. aeruginosa* QS-related genes. Relative mRNA expression of *lsaR* (**a**), *lasI* (**b**), *rhlR* (**c**), *rhlI* (**d**), and *pqsR* (**e**). P: *P. aeruginosa* (1 × 10^8^ CFU/mL); L: *P. aeruginosa* + *L. plantarum*; H: *P. aeruginosa* + honey; HL: *P. aeruginosa* + honey + *L. plantarum*. Values are represented as mean ± standard error (SEM) (n = 3). The ** indicates a statistically significant difference (*p* < 0.01), and **** indicates *p* < 0.001.

To evaluate the effect of honey–*L. plantarum* formulation on *S. aureus* and *P. aeruginosa* at the molecular level, candidate genes expression analysis (involved in biofilm formation and virulence production) was performed by real-time PCR. In contrast to the controls of untreated (S) and *L. plantarum* treated (L), Figure 4 shows that the honey–*L. plantarum* formulation treatment upregulated the expression level of biofilm regulation-related genes (*icaA*, *icaR*, *sarA*, *agrA*, and *sigB*) for *S. aureus* (*p* < 0.05). However, after honey treatment (H) and honey–*L. plantarum* formulation treatment (HL), relative expression of the five critical QS-regulated genes (*lasI*, *lasR*, *rhlI*, *rhlR*, and *pqsR*) for *P. aeruginosa* were downregulated (Figure 5).

### 3.5. The Synergistic Antibacterial Activity of Honey–L. plantarum Formulation May Depend on the Honey Promotion Growth of L. plantarum

**Figure 6:**
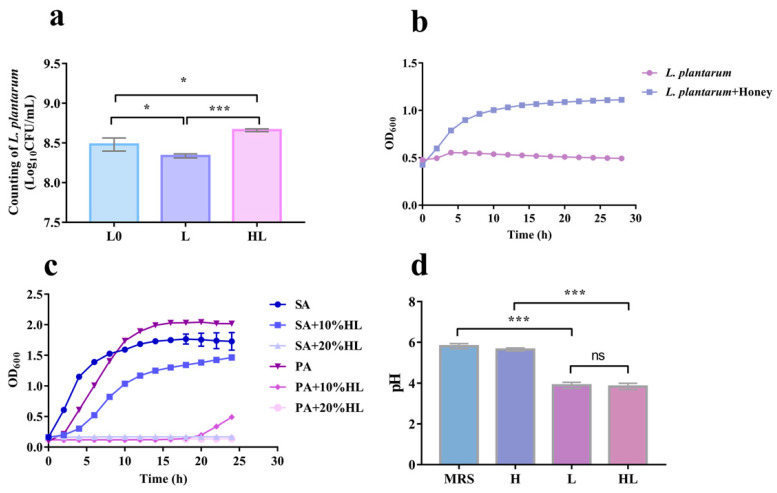
Growth effect of honey on *L. plantarum and* honey–*L. plantarum* culture supernatant to *S. aureus* and *P. aeruginosa.* (**a**) Growth counts of *L. plantarum* in a co-culture system with *S. aureus*, L0: the initial amount of *L. plantarum*, L: 24 h live count of *L. plantarum* in *S. aureus* co-culture, HL: 24 h live count of *L. plantarum* in honey and *S. aureus* co-culture; (**b**) growth curve of *L. plantarum*, (1 × 10^9^ CFU/mL), H (10%); (**c**) effect of honey–*L. plantarum* culture supernatant (CSF, *v*/*v*) on the growth of *S. aureus* (SA) *and P. aeruginosa* (PA) (1 × 10^7^ CFU/mL); (**d**) effect of honey–*L. plantarum* formulation on the pH of the culture medium for 24 h. H:MRS + honey (10%); L: MRS + *L. plantarum* (1 × 10^7^ CFU/mL); HL: MRS + *L. plantarum* (1 × 10^7^ CFU/mL)+ honey (10%). Data are expressed as mean ± standard error (SEM), * indicates a statistically significant difference (*p* < 0.05), *** indicates *p* < 0.001. The ^ns^ indicates none statistically significant difference (*p* > 0.05).

We sought to evaluate whether the synergistic antibacterial activity of honey–*L. plantarum* originates from the fact that honey has a promotive effect on the growth activity of *L. plantarum*. Thus, we first tested if the honey can promote the growth of *L. plantarum* and assayed the changes in the number of viable *L. plantarum* bacteria in the above antibacterial activity test. It showed that (Figure 6a) when there was no honey in the system (L) during the trial of the formulation’s inhibitory effect against *S. aureus*, the viable count of *L. plantarum* reduced by roughly 2% from the original level (L0). In contrast, the number increased by about 2% when honey was added to the cultures (HL), which showed that adding honey stimulated the growth of *L. plantarum* during the incubation. Then, the growth curves of *L. plantarum* alone and with honey in LB were further evaluated to investigate the growth-promoting effect of honey on *L. plantarum*. The experimental results showed that *L. plantarum* did not grow in the LB broth alone but grew and multiplied when co-cultured with honey (Figure 6b).

To find out if the metabolites of *L. plantarum* were antimicrobial, we also analyzed the honey–*L. plantarum* culture supernatant’s ability to inhibit *S. aureus* and *P. aeruginosa* growth, and we assessed the acid generation of *L. plantarum*. The results showed that honey–*L. plantarum* culture supernatant has an apparent inhibitory effect on *S. aureus* and *P. aeruginosa*. By measuring the growth curves of the two bacteria, as shown in Figure 6c, we found that 10% of honey–*L. plantarum* culture supernatant could significantly inhibit the growth of *S. aureus* and *P. aeruginosa*, and when the supernatant content reached 20%, it could completely inhibit the growth of both bacteria. Furthermore, we discovered that *L. plantarum* thrived in MRS medium for 24 h with a significant pH reduction in the medium, even if the pH reduction in the medium did not change noticeably when honey was present from that of *L. plantarum* alone (Figure 6d).

### 3.6. Honey–L. plantarum Formulation Promoted Wound Healing in Rats with Infections

**Figure 7:**
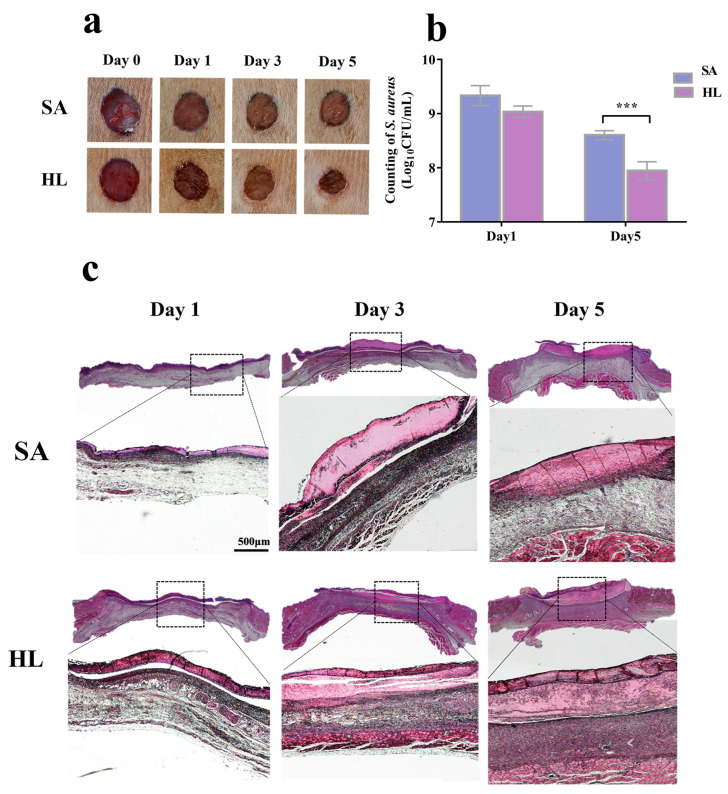
Effect of honey–*L. plantarum* formulation on wound healing. (**a**) Representative graphs of wounds on days 0, 1, 3, and 5; (**b**) wound bacterial residue counts; (**c**) H&E staining of wound tissue on days 1, 3, and 5; ×7.5 and ×40 magnification were used to observe. SA: a group of *S. aureus* infected control, with wounds infected by *S. aureus* of 1 × 10^8^ CFU/mL; HL: a group of treatment, with infected wounds treated by honey–*L. plantarum* formulation. Data are expressed as mean ± standard error (SEM), and the *** indicates a significant difference (*p* < 0.001) compared with the infected control (SA).

Infection of the wound by pathogenic bacteria can worsen the wound and slow its healing. For the viable count in the tissue around the wound (Figure 7a), after 5 days of continuous application of honey–*L. plantarum* formulation to the infected wound, the bacterial count in the honey–*L. plantarum* formulation group (HL) was about 0.7 log units less than that in the infected control group (SA). Moreover, the wound re-epithelialization and tissue granulation formation were assessed by H&E staining further to evaluate the wound healing effect of the honey–*L. plantarum* formulation. As shown in Figure 7b,c, many inflammatory cells were visible in the tissues of both the control and the treated groups 1 day after treatment. On the third day after treatment, a complete crust was formed on the wound surface in the treated group, with active growth of new connective tissue under the crust and less inflammatory exudation. In contrast, the control group’s inflammatory exudate and inflammatory damage were broader and more profound. On the fifth day, the new connective tissue in the treated group was quite active, and the wound had significantly decreased, meaning the wound was essentially healed. Though the wounds were covered in the control group, it was not easy to see the new connective tissue.

## 4. Discussion

Probiotic-derived biologics, such as organic acids, antimicrobial peptides, extracellular polysaccharides, and biosurfactants, have antibacterial and antibiotic film potential against many pathogens, such as *S. aureus*, *P. aeruginosa*, and *Salmonella enterica* [36,37,38]. Moreover, honey has antibacterial and anti-biofilm activity against *S. aureus* and *P. aeruginosa* [39,40]. Therefore, this study tried to shed light on the synergistic effect of *L. plantarum* and honey on pathogenic bacteria and the healing impact on infected wounds. Honey is considered a potential prebiotic due to its richness in oligosaccharides, and it has been shown that honey oligosaccharides can promote the growth of *Lactobacillus* and *Bifidobacterium*. In the present study, *L. plantarum* alone affected the growth of *S. aureus*, *P. aeruginosa*, and *E. coli*. Still, the combination of honey and *L. plantarum* significantly hurt pathogenic bacteria’s reproduction and biofilm formation.

Probiotics not only inhibit the activity of pathogenic bacteria and their adhesion to surfaces, but they also prevent the formation and survival of pathogenic biofilms, interfere with biofilm integrity, and eventually lead to biofilm eradication [41]. It has been shown that *L. plantarum* can inhibit the formation of pathogenic bacterial biofilms on catheters when applied to catheters [42]. To verify the effects of honey–*L. plantarum* formulation in inhibiting biofilm formation, both absorbance measurements and microscopic observations were performed. The crystal violet staining results showed that honey–*L. plantarum* formulation inhibited *S. aureus* and *P. aeruginosa* biofilm formation, and mature biofilms were also destroyed (Figure 2a,b). This was also confirmed in the fluorescence microscope, where we found that the honey–*L. plantarum* formulation effectively decreased the number of viable cells, increased the dead cell counts, and destroyed the dense and complete structure of the biofilm. Similarly, a recent study described that metabolites of *L. plantarum* inhibited the *Bacillus licheniformis* biofilm development by reducing live/dead cell counts, metabolic activity, and EPS content of biofilm adhered on stainless steel and glass surfaces [43].

Biofilm formation is an important factor affecting bacteria drug resistance. Studies have found that the expression of *ica*, *sarA*, and *agr* genes can regulate the secretion of virulence factors and the formation of biofilms in *S. aureus*. The biofilm distribution of *S. aureus* is mainly controlled by the *agr* genes, which can enhance biofilm dispersion and inhibit biofilm formation when strongly expressed. *sarA* is a global regulatory protein that affects the expression of many genes in S. aureus, including many involved in pathogenesis, making *sarA* a major virulence factor. *sarA* expression has consistently been shown to promote biofilm formation in *S. aureus* and *S. epidermidis* [44]. The *agr* system is normally involved in the transition from cell surface protein synthesis during exponential growth to the transition from toxin and degradation protein synthesis during the post-exponential to stable growth phase. The *agr* expression can reduce the ability of *S. aureus* to form biofilms [45]. In a recent study, *sarA* and *agrA* gene expression was downregulated in *S. aureus* after treatment with oxalic acid [46]. Similarly, *icaA*, *sarA,* and *sigB* expression levels were all downregulated, and *icaR* expression levels, a negative regulator of *icaA*, were upregulated after treatment of *S. aureus* with *Ginkgo biloba* exfoliating extract [23,29]. In the present study, the biofilm formation of *S. aureus* was significantly reduced and biofilm-related gene (*icrR*, *icrA*, *sarA,* and *agrA*) expression was upregulated after the honey–*L. plantarum* formulation treatment. Noteworthy is that our experiment showed an upregulation of transcription factor *sigB* gene expression (Figure 4e), which is in contrast to the current findings, even though the majority of research indicates that downregulation of the *sigB* can limit biofilm formation [29] We speculate that the interaction of honey with *L. plantarum* may have increased the abnormal expression of this gene, and the exact mechanism needs to be further explored and studied.

Additionally, a mechanism that enables microbial communication is called quorum sensing (QS). The primary function of QS is the regulation of critical cellular functions such as the production of virulence factors or the formation of biofilms [47]. There are many ways to inhibit the QS system, and the critical QS-related genes include *lasI*, *lasR*, *rhlI*, *rhlR*, and *pqsR*. In this study, we examined the relationship between the expression of QS-related genes and the formation of biofilms. The expression of *lasI*, *lasR*, *rhlI*, *rhlR*, and *pqsR* in *P. aeruginosa* considerably decreased after treatment with honey–*L. plantarum* formulation, which was consistent with the earlier finding [30,48]. Moreover, we discovered that although *L. plantarum* alone affected the expression of specific genes, including the upregulated *lasI* gene (Figure 5b) and downregulated *rhlR* gene (Figure 5c), the expression levels of these genes were all decreased when *L. plantarum* interacted with honey, which was in accordance with our expectations and crystalline violet staining experiments. Furthermore, there was no observable difference in gene expression levels between samples treated with honey alone (H) and those treated with honey–*L. plantarum* formulation (HL). This is likely because the interaction between *L. plantarum* and honey did not lead to the expected reduction in gene expression levels. After treatment with the honey–*L. plantarum* formulation, it is likely that the expression levels of these genes in *P. aeruginosa* biofilms decreased. Since this has crucial scientific significance for exploring the specific mechanisms by which the honey–*L. plantarum* formulation inhibited the formation of biofilms, more experimental confirmation is required.

A related investigation discovered that *L. plantarum* culture supernatants (CFSs) contain high levels of lactic acid, which is crucial for antibacterial and anti-biofilm resistances [13,49]. The production of extracellular proteases, cell surface hydrophobicity, EPS production, and hemolysis in *Streptococcus pyogenes* were found to be modulated by *L. plantarum* cell-free supernatants in a study on the anti-biofilm and anti-virulence of *L. plantarum* against *Streptococcus pyogenes*. The reduction in cell surface hydrophobicity affects the initial adhesion step, which is critical to the biofilm formation cascade and is considered to be the main cause of biofilm inhibition [50]. Interestingly, a recent study found that the cell-free supernatant extract of *Bifidobacterium thuringiensis* contained various compounds structurally similar to known anti-biofilm compounds, such as squalene, cinnamic acid derivatives, and eicosapentaene, with synergistic effects on *S. aureus* biofilms [51]. Such findings indicate that the antimicrobial effect of *L. plantarum* cell-free supernatant is mainly derived from the production of key antimicrobial and anti-biofilm substances by *L. plantarum* itself.

The primary mechanism of probiotic bacteria to inhibit pathogens through the production of some substances such as organic acids, hydrogen peroxide, low molecular weight antimicrobial substances, and bacteriocins has been investigated [52]. *L. plantarum* had strong metabolic activity and significantly inhibited the growth of pathogenic bacteria during co-cultivation with *Salmonella typhimurium*. The mechanism of action may be related to the lowering of the pH value of the medium by *L. plantarum* [53]. In this investigation, honey served as a good carbon source for the growth and reproduction of *L. plantarum* (Figure 6a,b). The greater quantity of *L. plantarum* increases the possibility of co-aggregation, which allows bacteria to aggregate and produce essential metabolic products such as organic acids, lowering the pH of the medium to more effectively exert its antibacterial effect (Figure 6d).

Furthermore, probiotics have an active role in preventing and healing infected wounds [54]. They interfere with the wound healing process by affecting collagen synthesis, amplifying the expression of tight junction proteins, enhancing the migration of keratin-forming cells, and stimulating proliferation. On the other hand, honey can not only treat bacterial infections but also work as a good wound dressing because of its antibacterial qualities brought on by the creation of hydrogen peroxide by the enzyme glucose oxidase in honey. Thus, we chose honey as one of the media in our antimicrobial formulation to assist *L. plantarum* in healing infected wounds. Our results confirmed the effective part of honey–*L. plantarum* in infected wounds in rats. Moreover, *L. plantarum* grew and multiplied in large numbers with the assistance of honey (Figure 6b), which may reduce the risk of pathogen adhesion to wound cells or the replacement of pathogens.

Local wound infections can significantly affect healing because bacteria prolong the inflammation and interfere with the epithelial formation, contraction, and collagen deposition [55]. During the inflammatory phase of wound healing, neutrophils and macrophages enter the wound site to release and secrete large amounts of enzymes and cytokines to remove and digest invading bacteria and cellular debris to prevent infection [56]. In the group treated with the honey–*L. plantarum* formulation, the number of bacteria in the wound tissue was significantly reduced. Moreover, in the pre-healing phase, inflammatory cells increased dramatically, indicating that the honey–*L. plantarum* formulation could regulate the number of inflammatory cells in the wound area and reduce the negative effect of wound infection on healing. Skin tissue repair aims to restore the barrier function of the skin, for which granulation tissue is required to replace the defect to form new connective tissue and epithelial wound closure is necessary to restore the physical barrier [57]. In our histological results, scar remodeling at the neoplastic epithelium was achieved by significant accumulation and thickening of granulation tissue after epithelial wound closure, starting from an early phase dominated by inflammation. The healing process in the honey–*L. plantarum* formula treatment group followed the mentioned pattern. These results indicate that the honey–*L. plantarum* formulation effectively promoted wound healing in infected rats, confirming its positive role in treating infected wounds.

**Figure 8:**
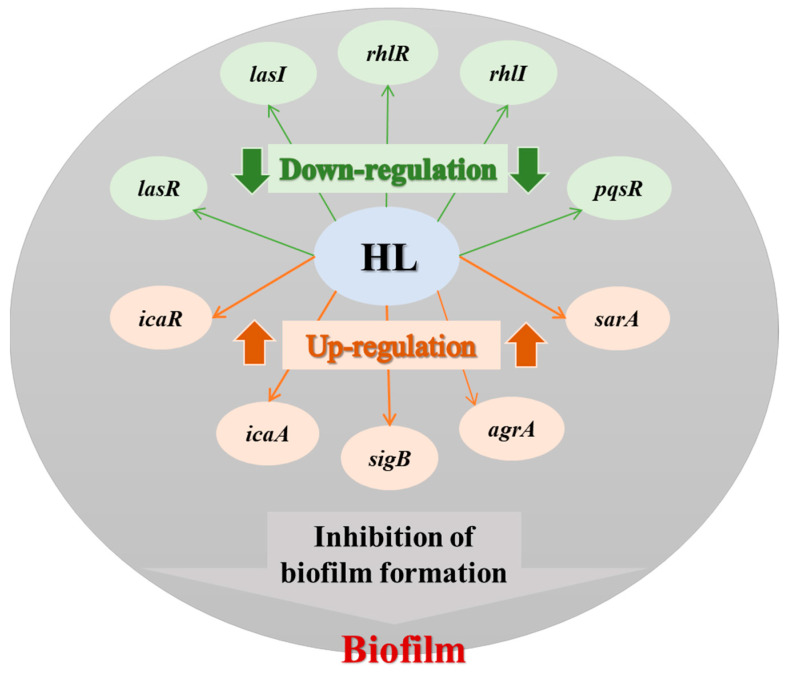
A possible inhibition graphic model of the honey*–L. plantarum* in gene regulation. HL: honey–*L. plantarum* formulation.

Based on the expression levels of quorum sensing key regulatory genes and biofilm-related genes, the possible mechanism of inhibition of biofilm formation by honey–*L. plantarum* formulation was deduced (Figure 8). The proposed graphical model is only a schematic and the exact role of the formula in the gene regulation mechanism needs further study.

## 5. Conclusions

In conclusion, in our study, the honey–*L. plantarum* formulation was used to investigate its antibacterial effect and mechanism against pathogenic bacteria and applied to a rat wound infection model. The formulation exhibited effective antibacterial activity in vivo and in vitro, and qRT-PCR results suggested that the honey*–L. plantarum* formulation may inhibit biofilm formation by regulating some genes related to biofilm formation. Importantly, this formulation could not only control pathogenic bacterial infections but also promote wound healing, playing a potential role in treating clinically relevant bacterial infections. Therefore, this study will provide a new practical reference for the clinical treatment of pathogenic bacterial infections, the promotion of wound healing, and changes in the management of pathogenic bacteria.

## Figures and Tables

**Table 1:** 3 × 3 factorial design levels and factors.

Factors	Level 1	Level 2	Level 3
X1 (Honey ratio, %, *v*/*v*)	10	20	30
X2 (*L. plantarum*, CFU/mL)	10^7^	10^8^	10^9^

**Table 2:** Gene-specific primers used in this study.

Gene	Forward Primer	Reverse Primer
*rpoB*	CAGCTGACGAAGAAGATAGCTATGT	ACTTCATCATCCATGAAACGACCAT
*icaA*	CTGGCGCAGTCAATACTATTTCGGGTGTCT	GACCTCCCAATGTTTCTGGAACCAACTCC
*icaR*	TGCTTTCAAATACCAACTTTCAAGA	ACGTTCAATTATCTAATACGCCTGA
*sigB*	AAGTGATTCGTAAGGACGTCT	TCGATAACTATAACCAAAGCCT
*agrA*	TGATAATCCTTATGAGGTGCTT	CACTGTGACTCGTAACGAAAA
*sarA*	CAAACAACCACAAGTTGTTAAAGC	TGTTTGCTTCAGTGATTCGTTT
*lasI*	CGATACCACTGGCCCCTACA	GGCTGAGTTCCCAGATGTGC
*lasR*	AGGAAGTGTTGCAGTGGTGC	GGAGGTCACACCGAACTTCC
*rhlI*	GTCTCGCCCTTGACCTTCTG	ATTCTGGTCCAGCCTGCAAT
*rhlR*	CGGGTGAAGGGAATCGTGTG	ACGGTTTGCGTAGCGAGATG
*pqsR*	CTGCTCACCGTATCGCAGAA	CGCCTGATCCCTTACATGCG
*GAPDH*	CACTCCAGCCGTTTCGAACT	CGGCTTGAACACCACCGTAT

**Table 3:** ANOVA table for the studied honey (H) and *L. plantarum* (L).

Source of Variation	Degrees of Freedom	Sum of Squares	Mean Square	*F*-Value	*p*-Value
Model	8	48.052	6.007	5115.985	<0.001
H (X_1_)	2	32.202	16.101	13,713.656	<0.001
L (X_2_)	2	5.095	2.548	2169.997	<0.001
H × L (X_12_)	4	10.755	2.689	2290.144	<0.001
Residual	18	0.021	0.001		
Cor Total	26	48.073			

**Table 4:** Honey and *L. plantarum* formulations and their antimicrobial activity.

Formulations	Honey (%)	*L. plantarum* (CFU/mL)	*S. aureus* (Log_10_CFU/mL)	Inhibition (%)
F0	0	0	9.60 ± 0.04	-
F1	10	10^7^	9.39 ± 0.01	2.20 ± 0.09
F2	10	10^8^	8.54 ± 0.04	11.04 ± 0.37
F3	10	10^9^	4.32 ± 0.12	55.05 ± 1.23
F4	20	10^7^	9.08 ± 0.02	5.40 ± 0.16
F5	20	10^8^	8.64 ± 0.03	10.01 ± 0.32
F6	20	10^9^	6.70 ± 0.10	30.26 ± 0.1.06
F7	30	10^7^	9.16 ± 0.01	4.62 ± 0.13
F8	30	10^8^	8.81 ± 0.02	8.25 ± 0.25
F9	30	10^9^	7.95 ± 0.05	17.23 ± 0.50

## Data Availability

The data that support the findings of this study are available from the corresponding author upon reasonable request.

## Supplementary Materials

The following supporting information can be downloaded at: https://www.mdpi.com/article/10.3390/pathogens12030501/s1, Figure S1: Scheme of the experimental design; Table S1: Antibiotic resistance in *S. aureus*, *P. aeruginosa* and *E. coli* [58].
