# Supplementary material for: Synergistic Inhibitory Effect of Honey and Lactobacillus plantarum on Pathogenic Bacteria and Their Promotion of Healing in Infected Wounds
Source: Pathogens. 2023 Mar 22;12(3):501. doi: 10.3390/pathogens12030501 (PMC10053434; doi:10.3390/pathogens12030501)
Supplement: Supplementary file 1 [file pathogens-12-00501-s001.zip › pathogens-2265616-supplementary.pdf]

# Supplementary material

# Synergistic Inhibitory Effect of Honey and *Lactobacillus plantarum* on Pathogenic Bacteria and Their Promotion of Healing in Infected Wounds

**Mei Li [1,†], Hong Xiao [1,†], Yongmei Su [2], Danlin Cheng [3], Yan Jia [1], Yingli Li [1], Qi Yin [1], Jieying Gao [1], Yong Tang [4,*] and Qunhua Bai [1,*]**

**Table S1.** Antibiotic resistance in *S. aureus*, *P. aeruginosa* and *E. coli*

| Antibiotic | *S. aureus* | *P. aeruginosa* | *E. coli* |
|---|---|---|---|
| Amikacin | I | I | I |
| Cefazolin | I | - | R |
| Ciprofloxacin | I | S | R |
| Penicillin | R | - | - |
| Gentamicin | I | I | I |
| Erythromycin | I | - | - |
| Ampicillin | R | - | R |
| Chloramphenicol | I | - | S |
| Cotrimoxazole | I | I | - |
| Norfloxacin | I | S | R |

*S* susceptible, *I* intermediate, *R* resistant[58]

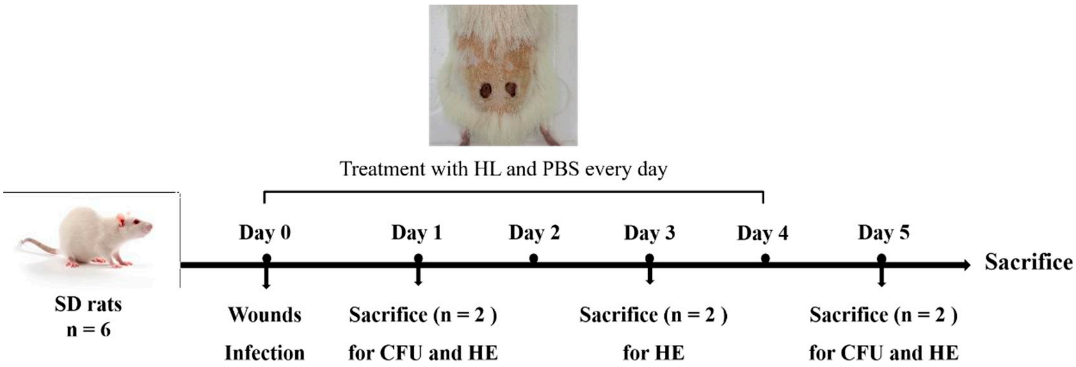


**Figure S1** Scheme of the experimental design

CFU: Count of viable bacteria in wound tissue; HE: Histological analysis.

## Reference


1. Cockerill, F.R.; Clinical and Laboratory Standards Institute. *Performance Standards for Antimicrobial Susceptibility Testing: Twenty-Third Informational Supplement; [... Provides Updated Tables for... M02-A11, M07-A9, and M11-A8]*; National Committee for Clinical Laboratory Standards: Wayne, PA, USA, 2013.
